# Carbonic Anhydrase 5 Regulates Acid-Base Homeostasis in Zebrafish

**DOI:** 10.1371/journal.pone.0039881

**Published:** 2012-06-22

**Authors:** Ruben Postel, Arnoud Sonnenberg

**Affiliations:** Division of Cell Biology, The Netherlands Cancer Institute, Amsterdam, The Netherlands; Vanderbilt University Medical Center, United States of America

## Abstract

The regulation of the acid-base balance in cells is essential for proper cellular homeostasis. Disturbed acid-base balance directly affects cellular physiology, which often results in various pathological conditions. In every living organism, the protein family of carbonic anhydrases regulate a broad variety of homeostatic processes. Here we describe the identification, mapping and cloning of a zebrafish *carbonic anhydrase 5* (*ca5*) mutation, *collapse of fins* (*cof*), which causes initially a collapse of the medial fins followed by necrosis and rapid degeneration of the embryo. These phenotypical characteristics can be mimicked in wild-type embryos by acetazolamide treatment, suggesting that CA5 activity in zebrafish is essential for a proper development. In addition we show that CA5 regulates acid-base balance during embryonic development, since lowering the pH can compensate for the loss of CA5 activity. Identification of selective modulators of CA5 activity could have a major impact on the development of new therapeutics involved in the treatment of a variety of disorders.

## Introduction

Maintaining proper homeostasis is essential for every living organism. Homeostatic imbalance directly affects cellular metabolism, which eventually leads to physiological defects and pathologic conditions. Carbonic anhydrases (CA) are zinc metalloenzymes that are present in prokaryotes and eukaryotes. They catalyze the reversible dehydration/hydration reaction of carbon dioxide (CO_2_ + H_2_O ↔ HCO_3_^−^+ H^+^) [1], [2]. CAs are involved in many physiological processes such as transport of carbon dioxide and bicarbonate between tissues, acid-base balance and biosynthetic reactions (glucogenesis, lipogenesis and ureagenesis) [3]. CAs are also important therapeutic targets, because of their involvement in various pathological conditions, such as glaucoma, obesity, some infectious diseases, cancer, epilepsy and osteoporosis [4] Therefore, many CA inhibitors and activators have been developed in order to treat these disorders [4]. Of the five different classes of CAs (α-εCA), vertebrates only express proteins of the α-CA class, which comprises 16 members that differ in their kinetic properties, tissue distribution, subcellular localization and their susceptibility to inhibitors [2], [4]–[12]. Whereas most CA isoforms are localized in the cytosol or associate with the plasma membrane, carbonic anhydrase 5 (CA5) is the only mitochondrial α-CA [13]. In mammals CA5 is encoded by two genes, *CA5A* and *CA5B* and whereas CA5A is expressed only in the liver, CA5B is widely expressed in many tissues [14]. Here we describe the mapping, cloning and characterization of a *ca5* mutant zebrafish (*collapse of fins*, *cof*) and show that CA5 is involved in regulating acid-base balance during embryonic development in zebrafish.

## Methods

### Zebrafish strains and Forward genetic screening

Adult fish were raised and maintained under standard laboratory conditions. Fish experiments were performed in accordance with institutional guidelines and as approved by the Animal Experimentation Committee of the Royal Netherlands Academy of Arts and Sciences. The *cof* mutant was identified during a forward genetic screen performed at the Hubrecht Institute, Utrecht, The Netherlands. N-Ethyl-N-nitroso-ureum (ENU) mutagenesis was performed as previously described for the creation of the Hubrecht Institute target selected mutagenesis library [15]. F1 progeny of mutagenised male fish were outcrossed to wild-type fish in order to produce approximately 300 F2 families, which were then intercrossed. F3 progeny were screened for epidermal integrity defects at 2–3 dpf. Meiotic mapping of the *collapse of fins* mutation was performed using standard simple sequence length polymorphisms (SSLP). SSLP primer sequences can be found on www.ensembl.org. Genotyping PCR and subsequent sequencing of the *ca5^T839A^* mutation on finclip DNA or DNA of single embryos was performed with the following primers: F: 5 -cggacagcaagacatctg-3′ and R: 5′-ttgtggatacacatccccatag-3′.

### Zebrafish embryo culturing

Embryos were raised in egg medium (60 μg/ml sea salt) pH 7. After 24 hpf dechorionated embryos were collected and placed in agarose-coated culture dishes with egg medium or 1x Danieau's medium (58 mM NaCl, 0.7 mM KCl, 0.4 mM MgSO_4_, 0.6 mM Ca [NO_3_]_2_ buffered at different pH with 10 mM Hepes.

### Acetazolamide treatment

Acetazolamide (Sigma) was dissolved in DMSO to a concentration of 0.5 M and diluted to a working concentration of 2.5 mM and 5 mM in egg- or Danieau's medium. Control embryos were treated with the same amount of DMSO solvent.

**Figure 1:**
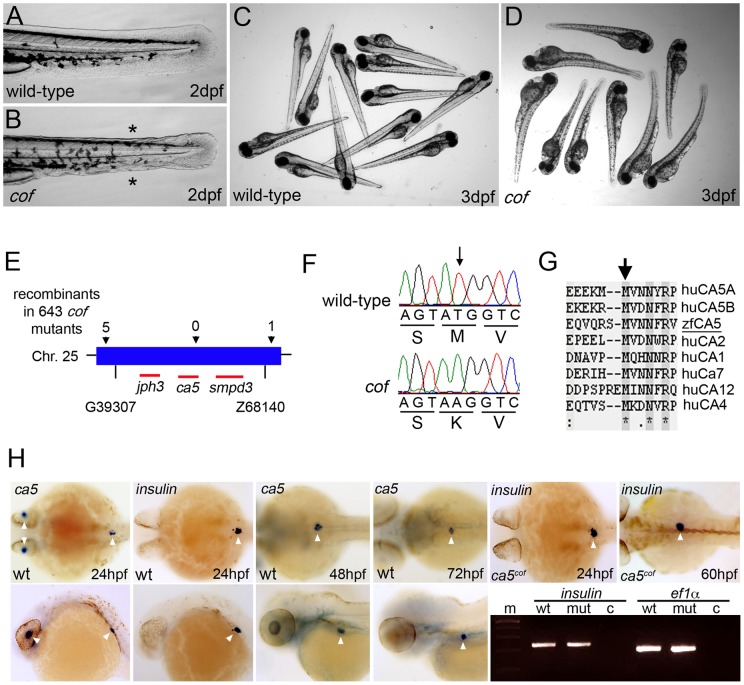
Characterization, mapping and cloning of the *cof* mutant. (A–D) Phenotypical comparison of wild-type and *cof* mutant embryos at 2 dpf (A, B) and 3 dpf (C, D). Asterisks mark the collapse of the medial fins in the *cof* mutant. (E) Summary of the linkage analysis and mapping of the *cof* locus at chromosome 25. The arrows mark the direction of the mutation. Red lines indicate the various transcripts in the genomic region. (F) Sequence chromatograms of wild-type and *cof* mutant cDNA. The corresponding amino acid residues are indicated below. (G) CA5 protein sequence alignment of zebrafish and human and other members of the human CA protein family. Arrow marks residue M280 that is substituted to a lysine in *cof* mutant embryos. (H) Detection of *ca5* mRNA in wild-type embryos at 24, 48 and 72 hpf by *in situ* hybridization. mRNA expression of *insulin* at 24 hpf marks the position of pancreatic β-cells. Upper panel shows dorsal view and lower panels lateral view. White arrowheads mark the expression in the lens and the pancreatic β-cells.

**Table 1:** Quantification of the injection experiments and the various treatments.

Phenotype (at 3 dpf)	% wild-type	% *cof* mutant
*cof* batch of embryos (n = 65)	81	19
100 pg full-length *ca5* RNA (n = 98)	99	1
100 pg *ca5^T839A^* RNA (n = 59)	83	17
untreated wild-type embryos (n = 81)	100	0
wild-type embryos treated with 5 mM AZA (n = 73)	10	90
wild-type embryos treated with 2.5 mM AZA (n = 65)	99	1
*cof* batch of embryos treated with 2.5 mM AZA (n = 58)	34	66
wild-type embryos raised at pH 5 (n = 67)	100	0
wild-type embryos raised at pH 7.6 (n = 67)	100	0
wild-type embryos raised at pH 10 (n = 67)	100	0
wild-type embryos +5mM AZA in pH 5 medium (n = 75)	77	23
wild-type embryos +5mM AZA in pH 7.6 medium (n = 84)	16	84
wild-type embryos +5mM AZA in pH 10 medium (n = 76)	4	96
*cof* batch of embryos in pH 5 medium (n = 81)	98	2
*cof* batch of embryos in pH 7.6 medium (n = 77)	82	18
*cof* batch of embryos in pH 10 medium (n = 68)	76	24

### *In situ* hybridisation, cDNA constructs and RNA synthesis

Whole mount *in situ* hybridization (ISH) was performed as described previously [16]. Embryos for ISH were fixed with 4% PFA/PBS and stored in 100% methanol. After ISH, embryos were cleared in methanol and mounted in benzylbenzoate/benzylalcohol (2:1) before images were taken. The following primers were used to produce the *ca5* cDNA fragment: F: 5′-tgcatccaatgtggcaggag-3′; R: 5′-ttgtgtctgactgcaggcaagg-3′ and the *insulin* cDNA fragment: F: 5′-ttggtcgtgtccagtgtaag-3′; R: 5′-tgcctctcttccttatcagc-3′. Fragments were cloned into the pCRII-TOPO vector (Invitrogen) and antisense dig-labelled probes were synthesised according to standard protocols. Full-length zebrafish *ca5* cDNA (MGC:171653; IMAGE:7448163) was derived by PCR on cDNA with the primers: F: 5′-gcgaattcaccatggtcacactgacagccat-3′ and R: 5′-gcctcgagttattccttagaggggg-3′ and cloned into the pCS2+ vector with EcoR1/Xho1. RNA was synthesised *in vitro* by using the SP6 mMessage mMachine kit (Ambion). The *ca5^T839A^* mutation was introduced using the QuickChange kit (Stratagene).

**Figure 2:**
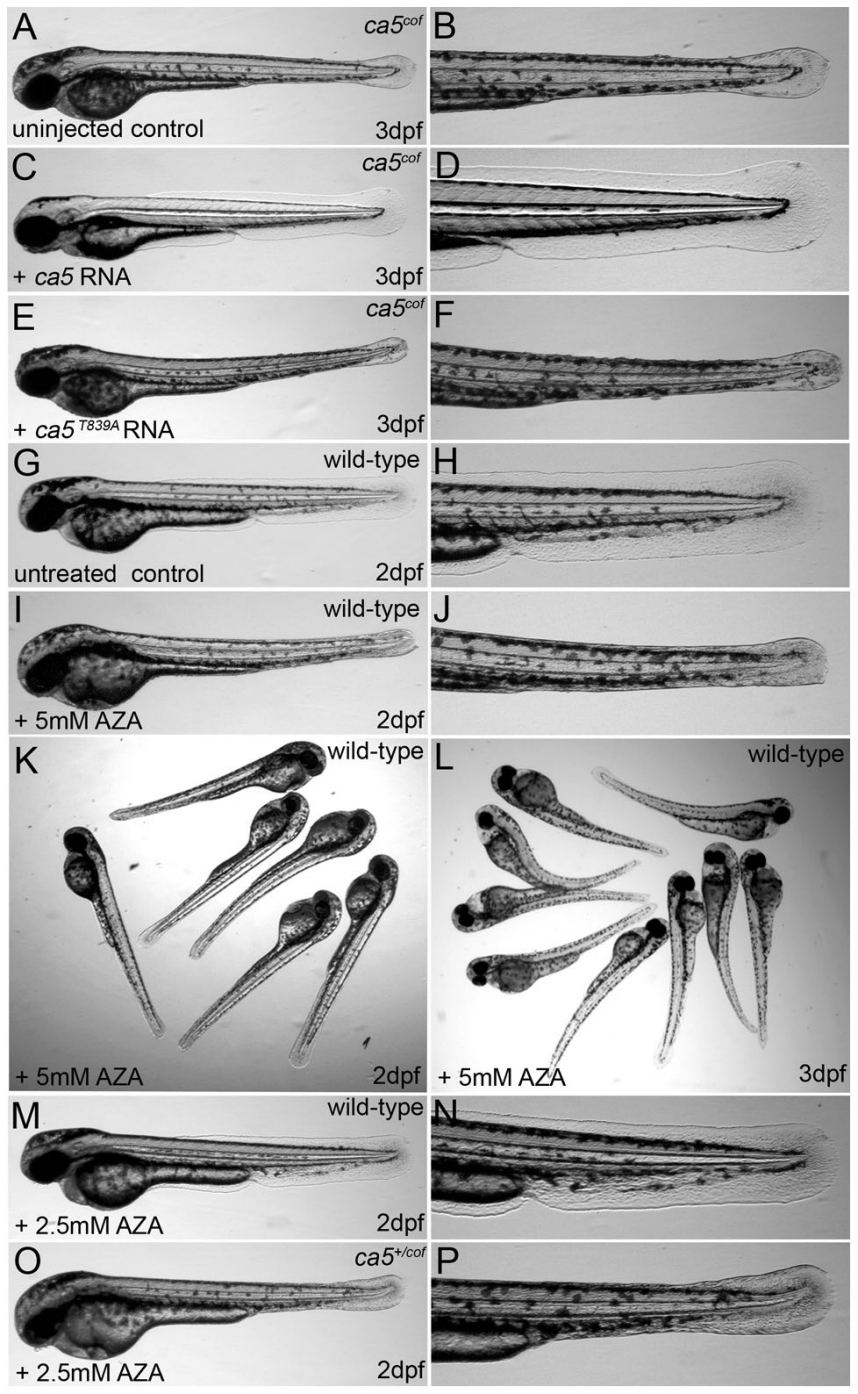
Rescue of the *ca5^cof^* mutant and acetazolamide treatment phenocopies the *cof* mutation in wild-type embryos. (A, B) *ca5^cof^* mutant embryos, (C–F) *ca5^cof^* mutant embryos injected with 100 pg full-length wild-type *ca5* (C, D) or 100 pg *ca5^T839A^* mutant RNA (E, F). (I–L) Wild-type embryos at 2 dpf (I–K) and 3 dpf (L) treated with 5 mM AZA. Treatment of wild-type and *cof* heterozygous embryos at 2 dpf with 2.5 mM AZA.

## Results

### A missense mutation in zebrafish *carbonic anhydrase 5* leads to collapse of the medial fins, heart failure and rapid degeneration of the zebrafish embryo

From a forward genetic screen in zebrafish we derived a mutant allele, *collapse of fins (cof)* that is characterized by defects of epidermal integrity and collapse of the medial fins at 2 days post-fertilization (dpf) (Figure 1A, B). During later stages of development, cardiac failure with edema and necrosis of the yolk-sac can be observed (Figure 1C, D), eventually leading to the rapid degeneration of the complete embryo at 4 dpf. The *cof* mutant phenotype is not fully penetrant, only 19% (instead of 25%) of the embryos can be phenotypically identified as a mutant in a batch of *cof* embryos (see Table 1). Meiotic mapping placed the *cof* allele on chromosome 25 between markers G39307 and z68140 (Figure 1E). Sequencing the open reading frames of the genes within the corresponding genomic interval revealed a T839A mutation in the coding region of the *ca5* gene (Figure 1F). *ca5* encodes for the zebrafish orthologue of CA5. The *ca5^T839A^* mutation results in an amino acid substitution of residue M280 to a lysine (Figure 1F). CA5 protein comparison analyses show that M280 is highly conserved across species and other members of the CA protein family (Figure 1G). The zebrafish genome contains only one *ca5* gene and comparison of the amino acid sequences reveals 31% identity between zfCA5 and huCA5A, and 40% between zfCA5 and huCA5B. In order to study the *ca5* mRNA expression, whole mount *in situ* hybridization was performed on wild-type embryos at various stages of development. This revealed *ca5* mRNA expression in the lens and in a specific part of the embryo that resembles the developing pancreas at 24 hpf (Figure 1H). Previous studies have identified human CA5B in the insulin-producing β-cells of the pancreas [17]. To verify the mRNA expression of *ca5* in the pancreatic β-cells in zebrafish, we compared *ca5* expression with the expression of *insulin,* a marker for the pancreatic β-cells at 24 hpf. Indeed *ca5* mRNA is localized at the same position as the *insulin* expressing cells (Figure 1H). During later stages of development, *ca5* remains expressed in the pancreas (Figure 1H). The expression of *insulin* mRNA in the *ca5^cof^* mutants was indistinguishable from that in wild-type embryos (Figure 1H), suggesting that β-cell development is not impaired in *ca5^cof^* mutants during development. This was confirmed by determining the level of *insulin* mRNA expression by PCR on cDNA of wild-type sibling and *ca5^cof^* mutant embryos at 60 hpf (Figure 1H). Although we observed a clear morphological defect in the medial fins of the *ca5^cof^* mutants, *ca5* expression could not be detected in the fin epidermis by *in situ* hybridisation.

**Figure 3:**
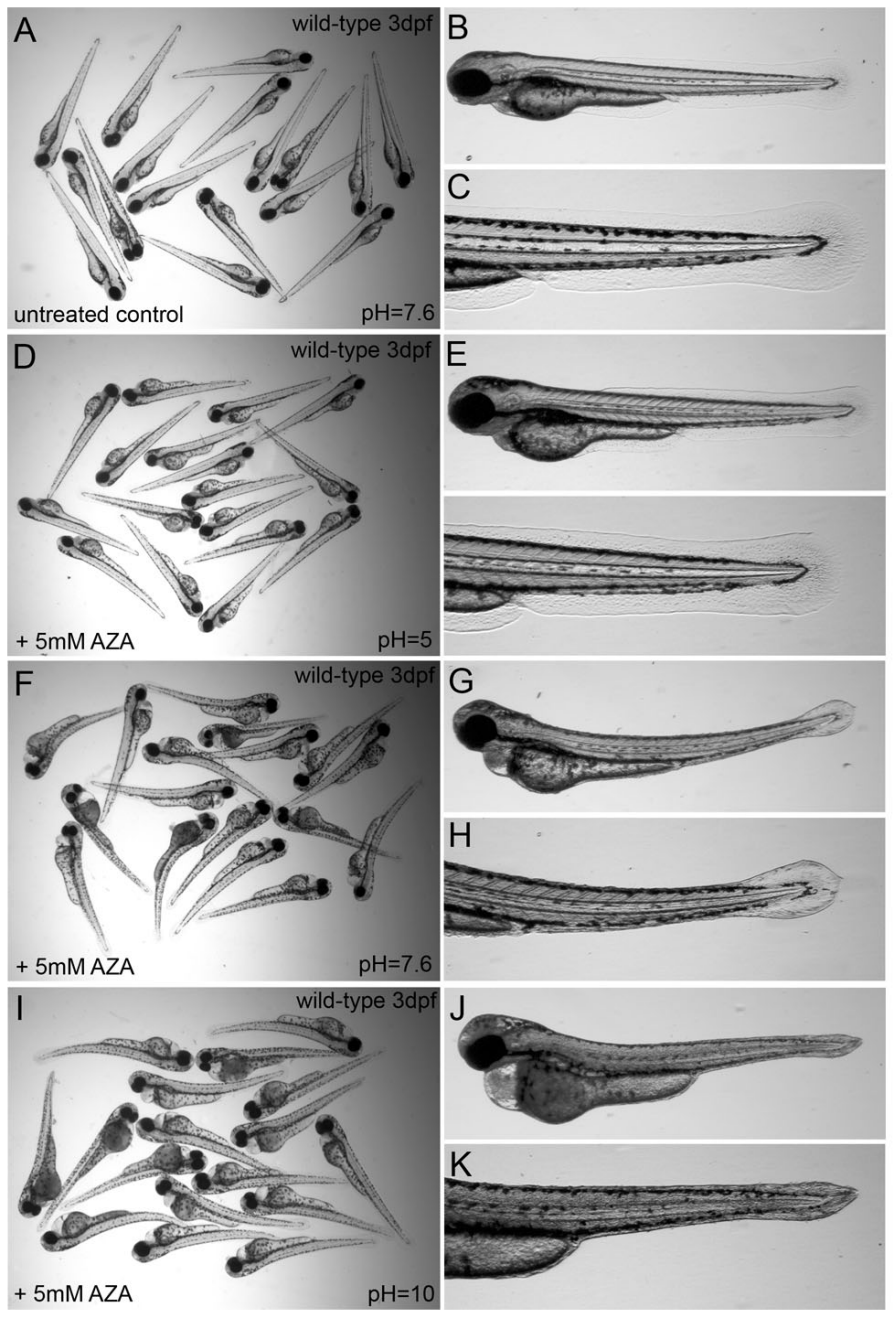
Low pH compensates for loss of carbonic anhydrase activity by acetazolamide treatment. (A–C) Untreated wild-type embryos, (D–L) wild-type embryos at 3 dpf treated in pH 5 (D–F), pH 7.6 (G–I), or pH 10 medium (J–L) with 5 mM AZA.

### Expression of CA5 rescues the *ca5^cof^* mutant phenotype and acetazolamide treatment in wild-type embryos phenocopies the *ca5^cof^* mutation

To examine whether the *ca5^T839A^* mutation in *ca5^cof^* mutant embryos causes the *ca5^cof^* mutant phenotype, we restored CA5 expression by injecting the full-length zebrafish *ca5* RNA. Injecting 100 pg *ca5* RNA rescued the *ca5^cof^* mutant phenotype completely, whereas it was not rescued after the injection of 100 pg mutant *ca5^T839A^* RNA (Figure 2A-F and Table 1).

In order to determine whether the *ca5^M280K^* substitution results in a reduced enzymatic activity of the CA5 protein we treated dechorionated wild-type embryos at 24 hpf with acetazolamide (AZA), a general CA inhibitor. Treatment with 5 mM AZA generated essentially a phenocopy of the *ca5^cof^* mutant fish including collapse of the medial fins, cardiac failure and necrosis of the yolk (Figure 2I-L and Table 1), ultimately leading to degeneration of the embryo. We verified the capacity of AZA to inhibit CA5 enzymatic activity by performing synergistic interaction experiments in *ca5^cof^* heterozygous sibling embryos. We treated wild-type embryos and a batch of *cof* embryos with suboptimal concentrations of AZA. The morphology of wild-type embryos treated with 2.5 mM AZA was not altered (Figure 2M, N and Table 1), however in the *cof* batch of embryos around 66% of the embryos showed the *ca5^cof^* mutant phenotype (Table 1). Sequencing revealed that a suboptimal dosage of AZA could induce the *cof* mutant phenotype in heterozygous embryos (Figure 2O, P and Table 1), whereas all homozygous wild-type sibling embryos were not affected. All this shows that inhibition of CA5 activity by AZA treatment during embryonic development can mimic the *ca5^cof^* mutant phenotype. Thus the *ca5^T839A^* missense mutation results in a severe reduction or loss of CA5 activity that initially leads to a collapse of the medial fins, followed by complete degeneration of the embryo.

### Low pH reduces the effects of acetazolamide treatment in wild-type embryos and rescues the *ca5^cof^* mutant phenotype

Carbonic anhydrases are also involved in the regulation of acid-base balance, also in fish [18]. Therefore we examined the effect of altered pH levels on the collapse of the medial fins in AZA-treated wild-type embryos and *ca5^cof^* mutant embryos. First, wild-type embryos were raised from 24 hpf onwards in Danieau's medium of pH 5, pH 7.6 or pH 10, containing 5 mM AZA. These experiments show that wild-type embryos are less susceptible to AZA, when cultured in pH 5 medium, compared to embryos cultured in pH 7.6 or pH 10 medium (Figure 3A-L). Furthermore, the *ca5^cof^* mutant phenotype can be rescued by raising mutant embryos in Danieau's medium of pH 5 (see Table1), suggesting that normally the increase in cellular pH during embryonic development is compensated by the activity of mitochondrial CA5 (Table 1). We could not observe any significant developmental defects when wild-type embryos were raised in medium of pH 5 or pH 10 (Table 1). All this shows that CA5 is involved in maintaining cellular acid-base balance during zebrafish embryonic development.

## Discussion

We show that defective CA5 activity in zebrafish results in a disturbed cellular acid-base balance, which leads to the collapse of the medial fins, heart failure and eventually degeneration of the complete embryo. We show that AZA, a general CA inhibitor, can copy the phenotype caused by the *ca5^cof^* mutation in wild-type embryos, suggesting that the T839A mutation results in the loss of CA5 enzymatic activity.

Human mitochondrial CA5 activity has been shown to be markedly elevated when the pH increases [19]. Thus loss or a reduced of CA5 activity results in an increase in cellular pH, which eventually leads to defects in cellular homeostasis. This is in accordance with our results in zebrafish that show that lowering the pH of the embryo medium can compensate for the loss of CA5 activity. In addition, the *cof* mutation is not fully penetrant when cultured in egg medium of pH 7 (∼19%), however an increase of the pH (pH 10) of the medium resulted in full penetrance of the mutation (∼24%) (Table 1), again showing that regulating acid-base balance is the major function of CA5 during zebrafish development.

Although the initial phenotypical defect is observed in the medial fins, *ca5* mRNA expression could only be detected in the pancreatic β-cells at 2 and 3 dpf. Defective CA5 function in the pancreatic β-cells cannot explain the medial fin defects and the rapid degeneration of *ca5^cof^* mutants. First of all, the level of *insulin* mRNA in the mutant embryos is not altered, suggesting that β-cell development is not impaired in *ca5^cof^* mutants. In addition, zebrafish mutants that lack pancreatic β-cells do not develop the phenotypical characteristics that we observe in the *ca5^cof^* mutant [20]. A plausible explanation for the severe medial fin defect and the rapid degeneration of the *ca5^cof^* mutant would be that CA5 is expressed at low levels in the epidermis. The defective epidermal acid-base balance, severely affects the epidermal barrier function, which results in rapid necrosis and degeneration of the embryo, especially in an aquatic environment. In fish several of the CA isoforms have been implicated in regulating physiological processes of the skin. For example, in a subtype of ionocytes of the skin and gills cytoplasmic CA regulates ionic exchange and acid-base balance [21]. However, knockdown of these cytoplasmic CA isoforms did not result in obvious morphological defects [21]. Here we observe a rapid degeneration of the complete embryo upon defective CA5 function, revealing that CA5 fulfils a major role in the regulation of cellular epidermal homeostasis, during development in zebrafish.

Although we did not see any effect on pancreatic β-cell development, we cannot rule out that defective CA5 function affects insulin secretion or could affect pancreatic β-cell development during later stages of development. Human CA5B is expressed in pancreatic β-cells and has been shown to provide bicarbonate for the first step of gluconeogenesis. It is therefore implicated in insulin secretion [17], [22]. Furthermore, inhibition of CA activity with AZA resulted in a strong inhibition of glucose-stimulated insulin secretion [17]. In the light of our findings, inhibition of insulin secretion in pancreatic β-cells after AZA treatment could be a secondary effect: defective acid-base balance causes impaired cellular homeostasis which leads to impaired insulin secretion.

Because CA5 is the only mitochondrial CA, it is an excellent pharmaceutical target. Currently many CA inhibitors and activators have been developed in order to treat a range of disorders [4]. Some of these compounds have been shown to inhibit or activate also the mitochondrial CA5 and are used in the clinic as anti-obesity or anti-epileptic drug [23]–[26]. However, pharmacological inhibitors that are selective for CA5 are currently not available.

In conclusion, in this study we report the identification of the first vertebrate *in vivo* model in which defective CA5 activity results in imbalanced cellular acid-base homeostasis. The fact that AZA treatment in wild-type embryos mimics the *ca5^cof^* mutant phenotype shows that zebrafish can be used as an easy and inexpensive *in vivo* model for screening and validating the functionality of novel CA5 modulators as potential therapeutics for a variety of diseases.
